# A Novel Synthetic Receptor-Based Immunoassay for Influenza Vaccine Quantification

**DOI:** 10.1371/journal.pone.0055428

**Published:** 2013-02-12

**Authors:** Anwar M. Hashem, Caroline Gravel, Aaron Farnsworth, Wei Zou, Michelle Lemieux, Kangwei Xu, Changgui Li, Junzhi Wang, Marie-France Goneau, Maria Merziotis, Runtao He, Michel Gilbert, Xuguang Li

**Affiliations:** 1 Centre for Vaccine Evaluation, Biologics and Genetic Therapies Directorate, Health Canada, Ottawa, Ontario, Canada; 2 Department of Microbiology, Faculty of Medicine, King Abdulaziz University, Jeddah, Saudi Arabia; 3 National Research Council Canada, Institute for Biological Sciences, Ottawa, Ontario, Canada; 4 National Institutes for Food and Drug Control, Beijing, People’s Republic of China; 5 National Microbiology Laboratory, Public Health Agency of Canada, Winnipeg, Manitoba, Canada; 6 Department of Biochemistry, Microbiology and Immunology, University of Ottawa, Ottawa, Ontario, Canada; University of Strathclyde, United Kingdom

## Abstract

Vaccination is the most effective prophylactic method for preventing influenza. Quantification of influenza vaccine antigens is critically important before the vaccine is used for human immunization. Currently the vaccine antigen quantification relies on hemagglutinin content quantification, the key antigenic component, by single radial immunodiffusion (SRID) assay. Due to the inherent disadvantages associated with the traditional SRID; i.e. low sensitivity, low throughput and need for annual reagents, several approaches have been proposed and investigated as alternatives. Yet, most alternative methods cannot distinguish native hemagglutinin from denatured form, making them less relevant to antigenic analyses. Here, we developed a quantitative immunoassay based on the sialic acid binding property of influenza vaccine antigens. Specifically, we chemically synthesized human and avian influenza virus receptors analogues, N-acetylneuraminic acid-2,6-lactose and N-acetylneuraminic acid-2,3-lactose derivatives with an azidopropyl aglycon, using α-2,6- and α-2,3-sialyltransferases, respectively. The azido group of the two sialyllactose-derivatives was reduced and conjugated to mouse serum albumin through a squarate linkage. We showed that the synthetic α-2,6- and α-2,3-receptors selectively bound to human and avian-derived hemagglutinins, respectively, forming the basis of a new, and robust assay for hemagglutinin quantification. Hemagglutinin treated at high temperature or low pH was measured differentially to untreated samples suggesting native conformation is dependent for optimal binding. Importantly, this receptor-based immunoassay showed excellent specificity and reproducibility, high precision, less turnaround time and significantly higher sensitivity and throughput compared with SRID in analyzing multiple influenza vaccines.

## Introduction

Influenza virus is a highly contagious respiratory pathogen which causes serious health effects worldwide. The continuous evolution of influenza virus due to the constant immune pressure as well as the occasional reassortment because of the segmented nature of viral genome are responsible for the occurrence of annual epidemics and periodic pandemics, respectively. The detrimental impact of influenza on human health can never be overstated. Indeed, there are up to 5 million cases of illness and up to 500,000 deaths associated with influenza every year [1], [2], [3].

Among the three known genera of influenza viruses, influenza A and B are mainly associated with human disease [1]. The two membrane-embedded surface glycoproteins, hemagglutinin (HA) and neuraminidase (NA) play crucial roles in the virus life cycle. While HA is involved in virus attachment to host cell surface receptors and subsequent entry into target cells by membrane fusion, NA is required for virus release from infected cells [4]. Although several studies have highlighted the role of NA in protection [5], [6], [7], [8], [9], [10], [11], HA is considered to be the main target for neutralizing antibodies upon natural infection or vaccination. There are at least 16 HA subtypes of influenza A virus that have been identified in aquatic birds [1], [12], but only strains from H1, H2 and H3 subtypes have become fully adapted to humans [12]. On the other hand, HA from influenza B viruses is classified into two lineages only, B/Victoria/2/87-like and B/Yamagata/16/88-like viruses, [13]. Influenza HA binds to host receptors, sialic acids (SA), which are linked to galactose. The linkage between the SA and the galactose determines the species preference of each HA. In particular, HA from human adapted viruses binds to SAα2,6-Gal, whereas avian HA preferentially binds to SAα2,3-Gal receptors [14], [15].

Typically, seasonal influenza vaccines are trivalent containing two influenza A (H1N1 and H3N2) strains and one influenza B strain [16], [17]. They predominantly induce HA strain-specific neutralizing antibodies. As indicated, the antigenic epitopes of the virus constantly evolve, making it necessary to annually update the virus seed strains and the reagents for vaccine potency assays. For more than 30 years, single radial immunodiffusion (SRID) has been used to standardize influenza vaccine potency and for vaccine lot release by most jurisdictions around the globe [18], [19]. SRID is a relatively simple, cheap and reproducible method which quantifies the antigenic HA in vaccine preparations against a homologous HA reference antigen [20], [21]. Several clinical trials have shown that SRID data correlate well with seroconversion and vaccine efficacy [22], [23], [24]. However, there are inherent disadvantages associated with SRID including the need to generate annual reference reagents (homologous antigens and corresponding subtype-specific antisera) by the WHO collaborating centres. This is a complex and time-consuming process representing a regulatory hurdle for timely release of vaccine lots as was witnessed during the H1N1 2009 pandemic [19]. In addition, it is not very sensitive for very low-dose vaccines [17], and it is a low throughput assay [19]. Therefore, the WHO and the European Medicines Agency (EMA) encourage the development of alternative methods for influenza vaccine standardization to complement or replace SRID.

Various research groups have investigated and continue to develop new approaches to address this issue. An early study showed that size exclusion-high performance liquid chromatography (SE-HPLC) is able to isolate the HA1 and HA2 subunits from vaccine preparations containing HA0 and provide a profile of protein components in vaccines according to protein size [25]. Several other groups also reported that reversed-phase HPLC (RP-HPLC) could be used to accurately separate and quantify absolute amounts of HA1 subunits from the three strains contained in trivalent formulations [26], [27], [28]. Moreover, Kapteyn et al. showed that RP-HPLC can be used with formaldehyde-inactivated egg or cell-derived vaccines, as well as subunit vaccines [29]. Interestingly, combining the two methods SE and RP-HPLC in two-dimensional (2D) HPLC resulted in higher sensitivity and selectivity for HA1 quantification [30]. Tandem mass spectrometry (MS)-based approaches were also investigated for characterization of influenza vaccines [31], [32], [33], [34]. Although these techniques have several advantages, especially in selectivity and sensitivity, they require expensive equipment and highly trained technical staff. During the 2009 pandemic, a few initiatives were undertaken to quantify HA content using some of these alternative methods for rapid release of vaccine lots. For example, the Chinese State Food and Drug Administration (SFDA) approved the use of SDS-PAGE and total protein measurement for HA content determination [35]. Moreover, a few clinical trials were based on potency values obtained by different manufacturers using HPLC as an alternative method in the US and Europe [19], [36]. While SDS-PAGE showed 88–120% consistency with SRID data [35], HPLC showed an underestimation of HA content in tested vaccines when they were re-evaluated using SRID [19], [36].

In addition to the reported physiochemical methods, alternative immunoassays for potency determination of influenza vaccine were also investigated. We previously generated universal anti-HA antibodies (Uni-1 Abs) targeting the highly conserved fusion peptide which bound to virtually all subtypes of HA [37], [38], [39]. Accordingly, they were used in multiple assay formats such as competitive ELISA (cELISA), slot blot and Western blot [40], [41] to analyze HA content in vaccines. More recently, Legastelois et al. reported two broadly reactive avian-glycan IgM mAbs [42] which were used in sandwich ELISA assays for HA quantification. These universal antibodies-based assays are very useful in quality control, especially for monovalent bulks and in-process manufacturing. However, they cannot be used with trivalent vaccines because they do not distinguish between the different HA subtypes. Although, an ELISA binding assay was previously developed based on binding of soluble horseradish peroxidase-labelled sialylglycoproteins to influenza viruses adsorbed to fetuin in microplates, this assay was mainly tested for receptor-binding properties and not evaluated for vaccine quantification [43].

SRID remains the most effective assay to quantify trivalent vaccines despite the development of several alternative assays, since it can presumably measure the native trimeric form of HA protein [44], which is known to be correlated with vaccine stability and immunogenicity [45], [46], [47], [48]. In addition, SRID is simple and does not require significant technological skill, making international comparisons or standardization relatively straightforward. On the other hand, most of the recently reported physiochemical and immunological methods do not differentiate between denatured and native protein structures, thus limiting their use in terms of vaccine lot release [19]. Clearly, there is still a need for additional approaches which would facilitate better vaccine quality control. Here, we report the development of an enzyme-linked immunosorbent assay (ELISA) based on the binding of HA to synthetic SA receptors. We present the detailed chemical synthesis process of the receptors and determined that they could distinguish the two types of HA (human vs. avian). When compared with SRID, the receptor–based ELISA (R-ELISA) was found to have higher sensitivity, higher throughput, less turnaround time and, most importantly, it is a stability-indicating assay.

## Materials and Methods

### Vaccines, Recombinant Proteins and Antibodies

Table 1 lists the vaccine strains, recombinant HA proteins and antibodies used in this study. The annual influenza reference antigens (H1N1, H3N2 and B) as well as the reference sheep antibodies were kindly provided by the National Institute for Biological Standards and Control (NIBSC), Potters Bar, U.K. The strain specific rabbit antibodies were purchased from Sino Biological Inc. (Beijing, P.R. China). The recombinant HAs were purchased from Proteins Sciences Corporation (Meriden, CT).

**Table 1:** Vaccines, recombinant HA proteins and antibodies used in this study.

**Human Vaccine (subtype)**	**Donor strains**
Influenza A (H1N1)	Influenza A/California/7/2009
Influenza A (H3N2)	Influenza A/Perth/16/2009(H3N2)-like*
Influenza B	Influenza B/Brisbane/60/2008
**Recombinant HA**	**Donor strains**
A/H1	Influenza A/New Caledonia/20/1999
A/H5	Influenza A/Vietnam/1203/2004
A/H7	Influenza A/Netherlands/219/03
B	Influenza B/Malaysia/2506/2004
**Antibodies**	**Immunogen**
Sheep anti-H1 A/California/7/2009(H1N1)	Influenza A/California/7/2009
Sheep anti-H3 A/Perth/16/2009(H3N2)-like	Influenza A/Wisconsin/15/2009
Sheep anti-HB B/Brisbane/60/2008	Influenza B/Brisbane/60/2008
Sheep anti-H1 A/New Caledonia/20/1999 (H1N1)	Influenza A/New Caledonia/20/1999
Sheep anti-H5 A/Vietnam/1203/2004 (H5N1)	Influenza A/Vietnam/1203/2004
Rabbit Uni-1 Abs (universal anti-HA antibody)	Influenza HA fusion peptide [37], [38], [39]
Rabbit anti-H1 A/California/7/2009(H1N1)	Influenza A/California/7/2009
Rabbit anti-H3 A/Victoria/210/2009(H3N2)	Influenza A/Victoria/210/2009
Rabbit anti-HB B/Brisbane/60/2008	Influenza B/Brisbane/60/2008

* Monovalent vaccine strain was A/Victoria/210/2009 (H3N2) and trivalent vaccine strain was A/Wisconsin/15/2009 (H3N2).

### Synthesis of the Avian and Human Influenza Receptors

#### Sialyltransferases

The avian receptor was synthesized using the *Campylobacter jejuni* Cst-I α-2,3-sialyltransferase (construct CST-06) which was expressed and purified as described before [49]. The human receptor was synthesized using the α-2,6-sialyltransferase from *Photobacterium* sp. JT-ISH-224 which was previously characterized [50], [51], [52]. A synthetic gene containing amino acids 110 to 514 of the α-2,6-sialyltransferase from *Photobacterium* sp. JT-ISH-224 (GenBank accession number BAF92026) was obtained from GenScript (Piscataway, NJ). The gene encoding the truncated α-2,6-sialyltransferase was transferred to plasmid pCWori+ to be expressed as a fusion protein containing the *Escherichia coli* maltose-binding protein (without the leader peptide) at the N-terminus (construct PDS-06). The fusion protein was expressed in *E. coli* AD202 (CGSC 7297) and purified on amylose resin according to the manufacturer’s instructions (New England Biolabs, Beverly, MA).

#### Synthesis of N-acetylneuraminic acid (NeuAc)-α-2-3-Gal-β-1,4-Glc-β-O-propylazide (avian receptor)

The a-2,3-sialyllactoside was synthesized as described previously [53] using the α-2,3-sialyltransferase CST-06 and azidopropyl lactoside [54].

#### Synthesis of N-acetylneuraminic acid (NeuAc)-α-2-6-Gal-β-1,4-Glc-β-O-propylazide (human receptor)

The reaction mixture included 107 mg of azidopropyl lactoside (10 mM final concentration), 14 mM CMP-NeuAc, 50 mM Mes pH 6 and 5 units of the α-2,6-sialyltransferase PDS-06. The reaction was incubated for 70 minutes at 37°C to ensure the completion of the reaction as revealed by thin layer chromatography (TLC) and detection by charring in 5% (v/v) H_2_SO_4_-RhOH. Purification was conducted on a Bio-Gel P-2 column using water as eluent, yielding 70 mg of a-2,6-sialyllactoside in lyophilized form.

#### Reduction of azide

To a solution of azidopropyl sialyllactoside (30 mg) in methanol (5 mL) was added 10% Pd-C (50% water, 10 mg). The mixture was stirred under hydrogen at balloon pressure for 20 minutes until the reaction was complete. The filtrate was concentrated to generate aminopropyl sialyllactoside (20 mg).

#### Activation of receptors

10 mg of 10% (w/w) Pd-C (as a powder containing 50% water) was added to a solution of 30 mg azidopropyl sialyllactoside in 5 mL of methanol. To a solution of aminopropyl sialyllactoside (20 mg) in phosphate buffered saline (0.8 g NaCl, 0.2 g KCl, 1.44 g Na_2_HPO_4_ and 0.24 g KH_2_PO_4_, pH 7.2) PBS-MeOH (1.6 mL, 5∶3) was added diethyl squarate (40 µL) at pH 7 adjusted using sat. NaHCO_3_. The reaction was monitored by TLC analysis. After completion of the reaction the mixture was loaded onto a Biogel P-2 column and monoethyl squarate derivatives were obtained (15 mg). Both monoethyl squarate intermediates gave similar characteristic proton spectra. ^1^H NMR (400 MHz, D_2_O) d 1.26–1.32 (m, 3H, C*H*_3_CH_2_O), 1.59 (dd, 1H, H3a, J = 12.4, 12.4 Hz), 1.88 (s, 3H, NAc), 2.56 (dd, 1H, H3e, J = 12.4, 4.4 Hz), 4.27 (d, 1H, H1, Gal, J = 8.0 Hz), 4.33 (d, 1H, H1, Glc, J = 8.0 Hz), 4.48 (q, 2H, CH_3_C*H*_2_O, J = 6.8 Hz).

#### Conjugation

Monoethylsquarate derivative (15 mg) and 30 mg murine serum albumin (Sigma-Aldrich Canada Ltd., Oakville, ON) was dissolved in 0.1 M Na_2_HPO_4_ (4 mL) at pH 9 adjusted using 0.1 M NaOH. The mixture was kept at room temperature for 30 hours. The conjugation was confirmed by HPLC analysis and the conjugates were purified on a Biogel A 0.5 column using PBS as eluent. The fractions were collected, dialyzed and lyophilized to generate the final conjugates (24 mg). The ratio of receptors to MSA was estimated by matrix-assisted laser desorption/ionization mass spectrometry (MALDI-MS) (see Table 2).

**Table 2:** Characterization of conjugates by MALDI-MS.

Conjugate	MW of receptor	MW of MSA	Average MW of conjugate	Receptor/MSA
**A (avian receptor) α-2,3-sialyllactoside-MSA**	770	65,859	79,646	18
**B (human receptor) α-2,6-sialyllactoside-MSA**	770	65,859	77,332	15

The ratio of receptors to MSA was estimated by mass spectrometry analysis. MSA has a molecular weight of 65,859 Dalton by MALDI-MS, whereas the molecular weights of the α-2,3- and α-2,6-sialyllactoside conjugates are averaged around 79,646 Da and 77,332 Da, respectively, indicating 18 molecules of α-2,3-sialyllactoside are attached to MSA, and 15 of α-2,6-sialyllactoside to MSA.

### Enzyme-linked Immunosorbent Assay (ELISA)

Indirect ELISA was performed as described previously with minor modifications [55]. Briefly, 96-well plates (Nunc, Mississauga, ON) were coated overnight at 4°C with 100 µL of 4 µg/mL of human (α-2,6-sialyllactoside-MSA) or avian (α-2,3-sialyllactoside-MSA) receptors in 0.05 M carbonate buffer (pH 9.6) per well. The plates were then washed 3 times with PBS containing 0.05% (v/v) Tween-20 (PBS-T), followed by blocking with 5% (w/v) skim milk in PBS-T for 1 hour at 37°C. After 3 additional washes with PBS-T buffer, 100 µL of vaccines were added in a half-log serial dilution starting from 10 µg/mL in PBS-T. The plates were then incubated at 37°C for 1 hour. After 6 washes with PBS-T buffer, reference sheep antibodies (1∶2000 dilution), strain-specific rabbit antibodies (1 µg/mL) or Uni-1 Abs (1∶2000 dilution) were added in PBS-T with 5% (w/v) skim milk and the plates were incubated at 37°C for 1 hour. After extensive washing, peroxidase-conjugated donkey anti-sheep IgG (Sigma-Aldrich Canada Ltd., Oakville, ON) or goat anti-rabbit IgG (GE Healthcare, Baie d’Urfe, QC) was added at 1∶2000 dilution as recommended by the supplier and plates were incubated for 1 hour at 37°C. After 6 washes with PBS-T, tetramethylbenzidine (*TMB*) *substrate (*Cell Signaling Technology, Inc. Danvers, MA) was added and incubated for 30 minutes for colorimetric development. The reaction was stopped with 0.16 M sulfuric acid and the absorbance was read spectrophotometrically at 450 nm to quantify the HA antigens bound to the receptors at each dilution. A reference homologous antigen was included on each ELISA plate. Vaccine concentrations were determined using parallel line analysis to compare absorbance from each vaccine to that of the reference standard in 4-parameter regression model using Combistats 4.0 software.

### Single-radial-immunodiffusion Assay (SRID)

HA content in the different monovalent hemagglutinin preparations or trivalent vaccines was determined using SRID assay as previously described [56]. Briefly, 1% (w/v) agarose gels were cast with the appropriate HA antiserum (NIBSC) and wells were punched into the solidified gels. Vaccine and control samples were treated for 20 minutes at room temperature with Zwittergent 3–14 (Calbiochem-Behring Corp., La Jolla, CA) at a final concentration of 1% (w/v). The samples were then diluted with 1% (w/v) Zwittergent 3–14, loaded into the wells and incubated for 24 hours at room temperature. Following incubation, the gels were washed, mounted onto Gelbond film (Lonza, Rockland, ME, USA), dried and stained with Coomassie Brilliant Blue. The dried gels were scanned and potency estimates were determined through either the comparison of ring diameters or the number of pixels contained in the surface area of each ring compared to the corresponding reference antigen. Each sample was tested at least three times at four dilutions.

### Thermal Treatment

Monovalent vaccines were incubated at 4°C, 50°C or 100°C for one hour. Then, HA content in the treated vaccines was measured by SRID and R-ELISA using the corresponding sheep or rabbit strain-specific antibodies.

### Low pH Treatment

The pH treatment was described previously [46]. Briefly, the pH of vaccine samples in PBS was altered by adding an appropriate volume of 0.1 M acetate buffer to reach the desired pH. After 1 hour at 37°C the reaction mixtures were re-neutralized to pH 7.2 with 0.1 M NaOH. HA content in the treated vaccines was measured by SRID and R-ELISA using the corresponding sheep or rabbit strain-specific antibodies.

## Results

### Enzymatic Sialylation of Azidopropyl Lactoside Selectively Produced α-2,3-sialyllactoside (Avian Receptor) and α-2,6-sialyllactoside (Human Receptor)

**Figure 1:**
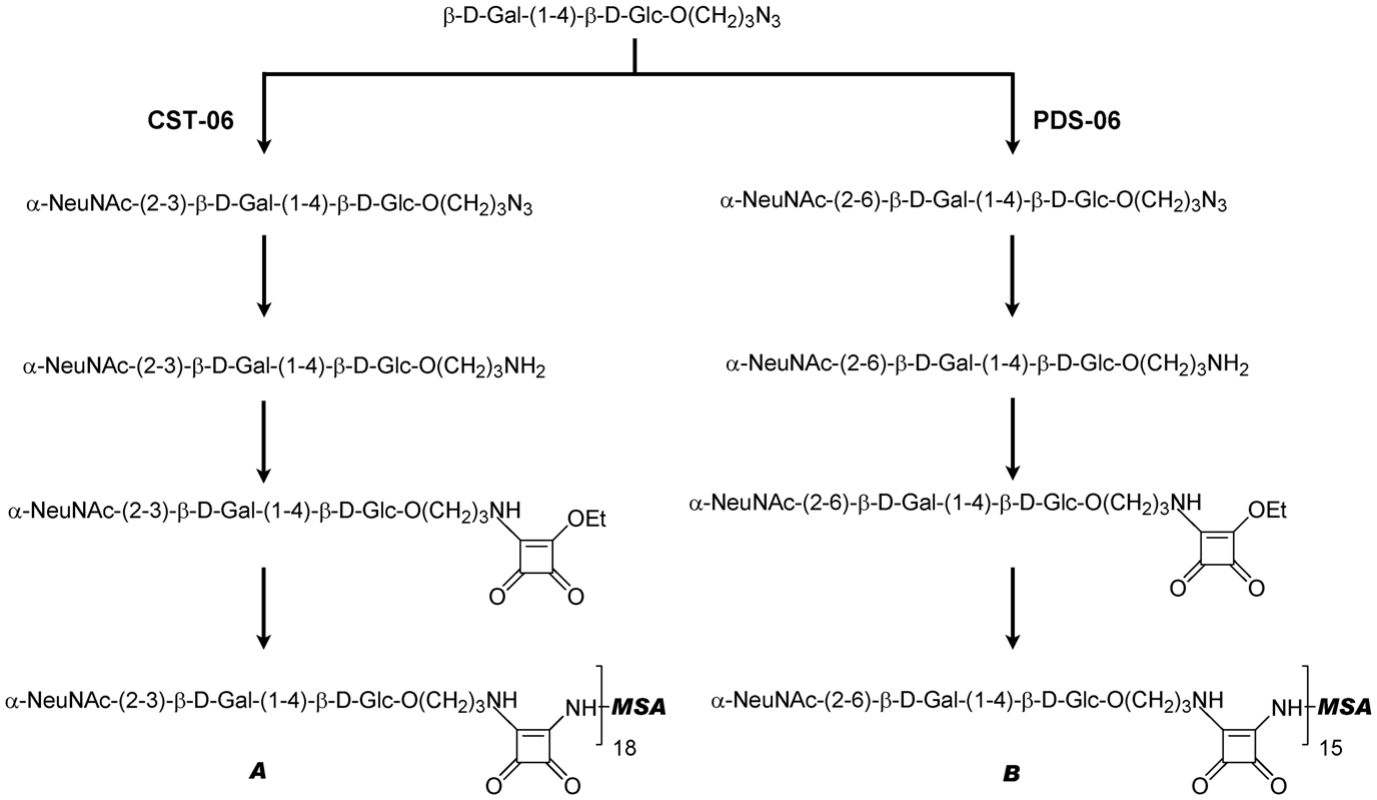
Synthesis of the avian and human influenza receptors and conjugation to mouse serum albumin (MSA). Schematic representation of steps involved in the synthesis and conjugation of the avian and human receptors. A is the avian receptor (α2,3-sialyllactoside-MSA) and B is the human receptor (α2,6-sialyllactoside-MSA). For the ratio of receptors to MSA as estimated by mass spectrometry analysis (MALDI-MS) see Table 2.

Azidopropyl lactoside (Gal-β-1,4-Glc-β-*O*-(CH_2_)_3_-N_3_) was used as the precursor for the synthesis of the HA receptors (Figure 1). Recombinant bacterial sialyltransferases were used to add the terminal sialic acid residues with the appropriate linkage specificity for each receptor. An α-2,3-sialyltransferase from *Campylobacter jejuni* [49] was used to synthesize the avian receptor and an α-2,6-sialyltransferase from *Photobacterium* sp. JT-ISH-224 [50] for the human receptor. The azido group of the aglycon of each synthetic receptor was reduced to an amine by catalytic hydrogenation (H_2_/Pd-C). In order to conjugate the sialyllactoside receptors to a protein carrier, e.g. mouse serum albumin (MSA), we employed diethyl squarate as a linker as previously described [57]. Diethyl squarate was first reacted with the amino group of the receptors at pH 7 to a monoethyl squarate intermediate. The conjugation between MSA and the monoethyl squarate intermediate was conducted at pH 9. The ratio of receptors to MSA was estimated by mass spectrometry analysis. MSA has a molecular weight of 65,859 Da by MALDI-MS, whereas the molecular weights of the α-2,3- and α-2,6-sialyllactoside conjugates are on average around 79,646 Da and 77,332 Da, respectively, indicating 18 molecules of α-2,3-sialyllactoside are attached to MSA, and 15 of α-2,6-sialyllactoside to MSA (Table 2).

### Synthetic Receptors Differentiate between Avian and Human HA

**Figure 2:**
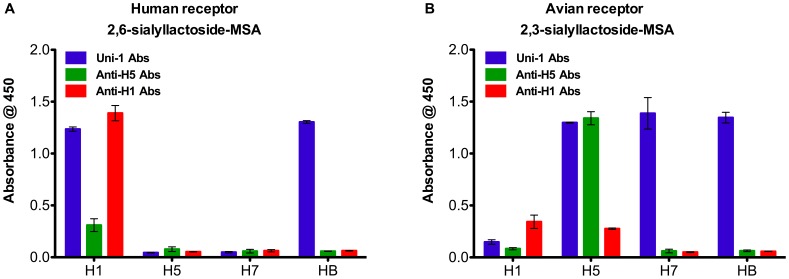
Binding specificity of human and avian synthetic receptors. ELISA plates were coated with either (**A**) the human α2,6-sialyllactoside-MSA receptor or (**B**) the avian α2,3-sialyllactoside-MSA receptor. Various types of recombinant HA proteins were tested. The binding of each recombinant HA to either receptor was detected with strain-specific antibodies and Uni-1 Abs which are universal antibodies against all HA proteins. Binding is shown as mean of absorbance from three experiments with error bars indicating the standard deviation.

To investigate the binding specificity of the synthetic receptors, we tested the binding of different recombinant HA proteins to either human or avian receptors in ELISA. As shown in Figure 2, human H1 (influenza A/New Caledonia/20/1999) protein showed classic human receptor preference compared to avian H5 (Influenza A/Vietnam/1203/2004) and H7 (Influenza A/Netherlands/219/03) proteins, which preferentially bound to the α2,3-sialyllactoside-MSA receptors only. In contrast, HA from influenza B/Malaysia/2506/2004 virus bound to both receptors. This is consistent with earlier demonstrations that influenza B viruses belonging to the Victoria-like lineage have binding specificities to both human and avian receptors [58]. These results confirm that the receptors are functional and show specificity in binding.

### R-ELISA Detects Native HA Trimer Rather than Denatured Forms

**Figure 3:**
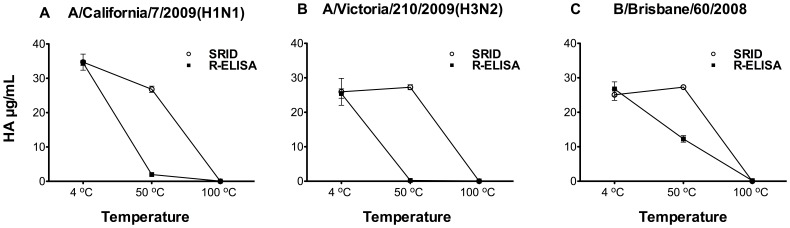
Effect of heat treatment on vaccine binding to the synthetic receptor. Influenza strains included in 2010–2011 vaccine (**A**) A/California/7/2009(H1N1), (**B**) A/Victoria/210/2009(H3N2) and (**C**) B/Brisbane/60/2008(Victoria-like) were incubated at 4°C, 50°C or 100°C for 1 hour, followed by measurements with both SRID and R-ELISA using the corresponding rabbit strain-specific antibodies. Each treatment was tested in triplicate/experiment and the experiment was repeated twice. Results are shown as mean of absorbance from two experiments with error bars indicating the standard deviation. (See also Figure S1).

Measurement of antigenic HA trimer rather than denatured HA in vaccines is a critical requirement in any alternative vaccine potency assay [19]. To investigate whether the R-ELISA could serve as a stability-indicating assay, we tested the effect of temperature on HA of the influenza strains included in 2010–2011 vaccine ((A/California/7/2009(H1N1), A/Victoria/210/2009(H3N2) and B/Brisbane/60/2008(Victoria-like)). As shown in Figure 3, heat-treatment of HA in these vaccines clearly reduced the binding between HA and the synthetic receptor as detected by both SRID and R-ELISA, with the latter being more sensitive in detecting temperature-induced structural changes. Similar results were also obtained when a different source of antibodies was used in R-ELISA (sheep anti-H1, H3 or type B HA) (Figure S1, supplementary materials).

**Figure 4:**
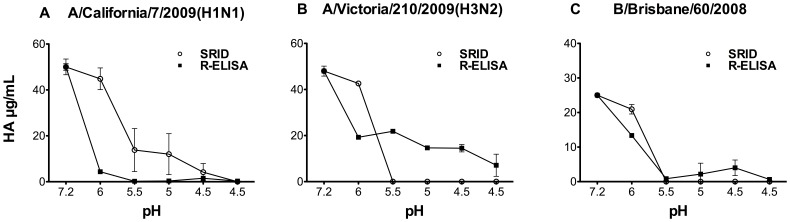
Effect of low pH treatment on vaccine captured by the synthetic receptor. Influenza strains included in 2010–2011 vaccine (**A**) A/California/7/2009(H1N1), (**B**) A/Victoria/210/2009(H3N2) and (**C**) B/Brisbane/60/2008(Victoria-like) were incubated at the indicated pH for 1 hour and then neutralized to pH 7.2 prior to measurement by both SRID and R-ELISA using the corresponding rabbit strain-specific antibodies. Each treatment was tested in triplicate/experiment and the experiment was repeated at least thrice. Results are shown as mean with error bars indicating the standard deviation.

Acidic conditions also result in irreversible conformational change of HA [59], [60]. Therefore we also evaluated the effect of low pH treatment on HA binding to the receptor. As shown in Figure 4, all HA samples tested (H1, H3 and type B) had substantially reduced binding to the synthetic receptors after exposure to low pH conditions; similar to SRID. Collectively, these results indicate that R-ELISA is capable of detecting trimeric HA protein in its native conformation.

### Antigen Specificity and Interference in the R-ELISA

**Figure 5:**
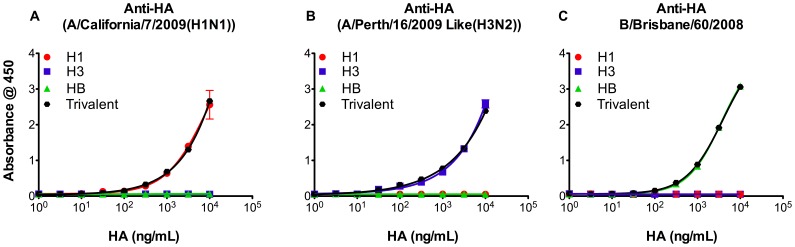
Specificity of synthetic receptor based ELISA. Influenza strains included in 2010–2011 vaccine (A/California/7/2009(H1N1), A/Victoria/210/2009(H3N2) and B/Brisbane/60/2008(Victoria-like)) (10 µg/ml) or a trivalent sample (containing 10 µg/ml from each strain) were measured by R-ELISA using (**A**) Rabbit anti-HA from Influenza A/California/7/2009(H1N1), (**B**) Rabbit anti-HA from Influenza A/Perth/16/2009 Like(H3N2) or (**C**) Rabbit anti-HA from Influenza B/Brisbane/60/2008. Each treatment was tested in triplicate/experiment and the experiment was repeated thrice. Results are shown as mean of absorbance with error bars indicating the standard deviation.

Because the seasonal vaccines are trivalent (H1N1, H3N2 and influenza B), we next investigated whether the R-ELISA is suitable for measurement of different HA antigens in the current trivalent vaccines. As shown in Figure 5, the strain-specific antibodies used to detect each specific virus showed no detectable cross-reactivity. More importantly, binding curves obtained from each antigen in monovalent vaccine were very similar to those in the trivalent preparations, suggesting that this assay can accurately quantify specific HA strains in either monovalent or trivalent formulations. Furthermore, we tested the effect of egg or mammalian cell adaptation on binding of vaccines to the human receptors. This experiment showed that both sources can be used in our R-ELISA (Figure S2, supplementary materials).

### Precision and Curve Fitting of R-ELISA

**Figure 6:**
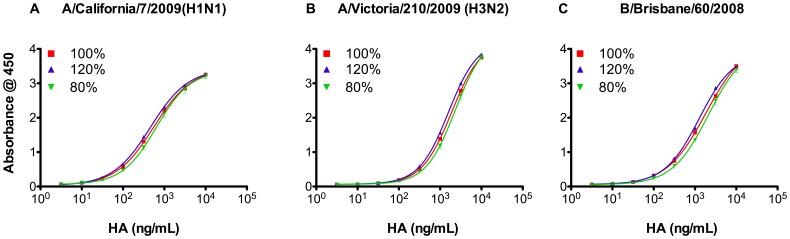
Precision of synthetic receptor based ELISA. Three expected ratios (80%, 100% and 120%) of the HA from each influenza reference strain in the 2010–2011 vaccine (**A**) A/California/7/2009(H1N1), (**B**) A/Victoria/210/2009(H3N2) and (**C**) B/Brisbane/60/2008(Victoria-like). Vaccine samples with known amounts of HA (pre-determined by SRID) were employed in spiking and recovery assays using strain-specific rabbit antibodies. Each sample was tested in triplicate. Data are shown as mean ± SD from two experiments (see also Figure S3). For HA concentrations obtained from each strain at each ratio see Table 3.

Known amounts of HA (pre-determined by SRID) were employed in spiking and recovery assays to determine the precision of the R-ELISA. For each strain, three expected ratios of the reference concentration were employed (80%, 100% and 120%). As shown in Figure 6 and Figure S3 (supplementary materials), the dose response curves for the three ratios satisfy the 4-PL model assumptions (i.e., regression, linearity and parallelism). Most importantly, the HA concentrations obtained from each strain were very close to the expected ratios (Table 3). Furthermore, these data clearly show the high sensitivity of the R-ELISA for very low vaccine concentrations with detection range of (0.1–10 µg/mL).

**Table 3:** Spiking and recovery.

Observed Concentration (µg/ml)	Expected Concentration (µg/ml)	Recovery (%)
**Influenza A/California/7/2009(H1N1) Vaccine**		
10.5±1.1	10	105.3±10.8
12.9±1.6	12	107.6±13.2
8.0±0.5	8	100.3±5.9
**Influenza A/Perth/16/2009(H3N2)-like**		
9.1±0.6	10	90.7±6.5
11.4±1.1	12	95.2±9.1
7.9±0.4	8	98.5±5.0
**Influenza B/Brisbane/60/2008 Vaccine**		
9.7±1.4	10	97.2±13.6
11.8±1.8	12	98.3±14.8
8.0±1.5	8	99.5±18.4

Each sample was tested in triplicate. Data are shown as mean +/− SD from two experiments.

HA concentrations were obtained using strain-specific rabbit antibodies.

### Quantification of HA Protein Content in Influenza Vaccine Using R-ELISA and SRID

We first conducted experiments to compare inter-assay variability between R-ELISA and SRID. In these experiments, samples were tested in triplicates at each dilution and each experiment was repeated 5 times. We found that in terms of variability, as suggested by CV, R-ELISA is performing at least as well as SRID. Interestingly, in the case of A/Victoria/210/2009 (H3N2) the HA amounts obtained by R-ELISA were much closer to predicted HA concentration (Table 4). We next used the R-ELISA to quantify HA in both multiple monovalent and trivalent samples in parallel with SRID against the same international reference antigens. As shown in Table 5, in comparison to the values determined by SRID, values obtained by R-ELISA were again very close to expected values, confirming that R-ELISA can be a viable alternative assay for HA quantifications.

**Table 4:** Intra-assay variation.

Monovalent Vaccine	Expected Concentration (µg/ml)	Observed Concentration (µg/ml)		Coefficient of Variation(CV)	
		SRID	R-ELISA	SRID	R-ELISA
**A/California/7/2009(H1N1)**	37.0	33.07±3.78	33.69±2.34	11.42%	6.96%
**A/Victoria/210/2009(H3N2)**	26.0	21.69±1.27	25.03±1.53	5.9%	6.1%
**B/Brisbane/60/2008**	27.0	24.47±1.76	25.62±1.73	7.2%	6.76%

Each sample was tested in triplicate. Data are shown as mean ± SD from five experiments.

**Table 5:** Quantification of HA in vaccines.

Influenza Strain	Vaccine Type	Expected Concentration (µg/ml)¶	Observed Concentration (µg/ml)	
			SRID	R-ELISA
**A/California/7/2009(H1N1)**	Monovalent 1	37.0	33.3 (29.3–37.4)	33.7 (28.7–39.6)
	Monovalent 2		33.4 (29.2–37.8)	34.6 (29.4–40.6)
	Monovalent 3		37.4 (33.2–42)	35.2 (30–41)
	Trivalent 1	30.0	41.7 (37.6–47.2)	40.5 (39–42)
	Trivalent 2		35.5 (31.4–41.1)	43.4 (41.7–45.1)
	Trivalent 3		39.7 (37.7–41.9)	25.5 (24.2–26.9)
**A/Perth/16/2009(H3N2)-like** *	Monovalent 1	26.0	22.1 (18.8–25.5)	24.1 (22.6–25.6)
	Monovalent 2		21.8 (18.7–24.8)	26.8 (25.1–28.6)
	Monovalent 3		19.9 (16.6–23.2)	25.4 (23.8–27)
	Trivalent 1	30.0	46.2 (43.6–49.3)	33.4 (32.5–34.4)
	Trivalent 2		43.6 (39.2–49.7)	35.6 (34.6–36.6)
	Trivalent 3		41.8 (38.1–46.9)	34.4 (33.2–35.7)
**B/Brisbane/60/2008**	Monovalent 1	27.0	26.1 (24.8–27.4)	28.9 (27.1–30.7)
	Monovalent 2		23.2 (21.9–24.4)	24.8 (23.4–26.3)
	Monovalent 3		25.9 (24.6–27.2)	26.6 (25.1–28.2)
	Trivalent 1	30.0	32.8 (30.5–35.5)	28.4 (27.2–29.6)
	Trivalent 2		36.3 (33.6–39.6)	27.4 (26.8–27.9)
	Trivalent 3		36.0 (34.4–37.9)	31.7 (30.8–32.7)

Each sample was tested in triplicate.

Data are shown as mean (95% Confidence interval).

* Monovalent vaccine strain was A/Victoria/210/2009(H3N2) and trivalent vaccine strain was A/Wisconsin/15/2009 (H3N2).

¶ Expected concentration is labeled values initially determined by SRID.

## Discussion

A variety of alternative assays have been developed for the potency determination of influenza vaccines since SRID is a tedious procedure and needs annually updated strain-specific antigens and antibodies as reference reagents. However, SRID remains useful primarily because it is sensitive to conformational change in the HA protein trimer and can therefore be used for stability analyses. Most of the reported alternative assays, particularly physiochemical assays, cannot determine the stability of the vaccines due to the use of denaturing conditions for protein separation or trypsin-cleavage of the proteins. Therefore, an alternative assay for potency determination of influenza vaccine would preferably be quantitative in a high throughput fashion and capable of analyzing trivalent vaccines and determining vaccine stability.

The R-ELISA reported here employs synthetic receptors to specifically capture correctly folded HA proteins followed by detection using strain-specific antibodies. The NA activity in the vaccine products does not appear to significantly affect the binding between HA and the synthetic receptor as the data from R-ELISA are comparable to that obtained from SRID. Our comparative studies revealed that R-ELISA performs at least as well as SRID in precision and reproducibility. Moreover, the use of 96-well ELISA plates facilitates high-throughput handling of samples and automation. It is of note that, in addition to its suitability for determining each viral HA component in trivalent vaccines, R-ELISA is more sensitive than SRID in detecting HA conformation changes induced by heating and at least as good as SRID in low pH conditions, an observation which needs to be better understood. However, the decreased signal in R-ELISA following lower pH treatment of the vaccine (Fig. 4) is mostly due to that the acidic pH-treated HA had lost its binding to the receptor not due to a weakened detection by the anti-HA polyclonal antibodies (Figure S4, supplementary materials).The R-ELISA may be more sensitive to changes in the native structure of HA because it is based on binding between the receptor and HA in the absence of a detergent (Zwittergent), whereas SRID is based on antigen-antibody precipitation in the presence of high concentration of detergent (usually 1% zwittergent). It needs to be mentioned that the use of 1% of Zwittergent is necessary in SRID to disperse samples, thereby allowing antigen diffusion in agarose. Yet, there is no such treatment of samples in R-ELISA, which might more accurately reflect the native conformation of the HA protein. Furthermore, the decreased absorbance reading following heat treatment in R-ELISA is suggestive of a loss in HA binding to the receptor. While the difference in HA contents between SRID and R-ELISA following heat treatment, particularly at 50°C, needs to be further investigated (Fig. 3), the purpose of this experiment is to examine whether heating can affect the HA binding to receptors. Moreover, it should be noted that future animal studies could be considered to verify the significance of HA contents obtained from R-ELISA following heat treatment of samples.

While the synthetic receptors with α-2,6- and α-2,3-linked sialic acids demonstrated excellent selectivity by binding to human and avian-derived HAs, respectively, this study is focused on characterization of the α-2,6 receptor-based R-ELISA for the analysis of human vaccines. We did not conduct full characterization of the α-2,3 receptor since sufficient avian vaccine samples were not available for assay validation beyond the specificity determination of binding using recombinant human and avian HA (Figure 2). Nevertheless, the selective binding of α-2,3 receptors to recombinant avian HA (H5 and H7) indicates the potential utility of this type of assay for stability tests of avian influenza vaccines, which are being developed or considered by numerous vaccine developers as a strategy to confront future pandemics through stockpiling candidate vaccines derived from avian viruses.

The R-ELISA reported here depends on the availability of the synthetic (N-acetylneuraminic acid-2,6-lactose) receptors which represents a technical challenge. However they can be readily synthesized as required using the protocol described here. Moreover, it would be interesting to investigate the potential lactoseamine derivatives in this R-ELISA platform as they might show better binding to influenza viruses [61]. Like any new assay, the R-ELISA still requires vigorous international validation for its suitability as a routine influenza vaccine quality control method. Yet, this assay differs from other previously reported alternative assays for influenza potency in that it is a stability-indicating immunoassay, which is an important requirement for assaying vaccine potency.

### Conclusions

Recently, several approaches have been proposed and investigated as alternatives to replace the traditional SRID method for HA quantification in vaccines. However, despite all of the inherent disadvantages (i.e. low sensitivity, low throughput, need for annual reagents), SRID remains the method of choice for vaccine potency determination since the majority of the previously reported assays cannot distinguish the native or antigenic HA from the denatured form of this protein. The influenza synthetic receptor-based ELISA (R-ELISA) developed here not only selectively quantify native HA trimers but also distinguishes itself from the traditional SRID assay in terms of higher throughput, greater sensitivity and shorter turnaround time.

## Supporting Information

Figure S1 **Effect of heat treatment on vaccine binding to the synthetic receptor.** Influenza strains included in 2010–2011 vaccine **(A)** A/California/7/2009(H1N1), **(B)** A/Victoria/210/2009(H3N2) and **(C)** B/Brisbane/60/2008(Victoria-like) were incubated at 4°C, 50°C or 100°C for 1 hour then measured by both SRID and R-ELISA using the corresponding sheep strain-specific antibodies. Each treatment was tested in triplicates and experiment was repeated twice. Results are shown as mean of absorbance and error bars indicate the standard deviation.
(TIFF)

Figure S2 **Binding of egg and cell-derived vaccines.** Binding of egg or cell-derived influenza A/California/7/2009(H1N1) strain starting at 10 µg/ml was tested by the R-ELISA using the strain-specific **(A)** Sheep anti-HA or **(B)** Rabbit anti-HA Abs. Each treatment was tested in triplicates and experiment was repeated twice. Results are shown as mean of absorbance and error bars indicate the standard deviation.
(TIFF)

Figure S3 **Precision of synthetic receptor based ELISA.** Three expected ratios (80%, 100% and 120%) of the HA from each influenza reference strain in the 2010–2011 vaccine **(A)** A/California/7/2009(H1N1), **(B)** A/Perth/16/2009(H3N2)-like and **(C)** B/Brisbane/60/2008(Victoria-like) as determined by the SRID were employed in spiking and recovery assay using NIBSC sheep antisera. Each sample was tested in triplicates. Data are shown as mean +/− SD from two experiments.
(TIFF)

Figure S4 **pH 6.0 treatment of vaccine does not result in a loss of binding between the vaccine and the polyclonal antibodies.** Vaccine sample (H1N1) were treated with lower pH (6.0) and then neutralized to pH 7.2. The samples were then coated to ELISA plates, followed by detection with the same polyclonal Ab used in R-ELISA. The results show that pH 6.0 did not cause a loss of HA binding to the polyclonal Ab. N.B.: when the universal antibody Uni-1 was used, the binding is generally weak in this direct ELISA although pH6.0 does result in a slightly better binding. In contrast, binding between receptor to HA resulted in a better exposure of the fusion peptide epitope which can be detected by Uni-1 as demonstrated by high OD values in Fig. 1. Collectively, these data (Fig. 4 & Figure S4) suggest that following pH 6.0 treatment of vaccine, the significantly decreased signal (Fig. 4A) is due to treatment of HA resulted in a conformational change, causing a substantially weakened capture by the receptor.
(TIF)
