# Supplementary figures and images for: A Novel Synthetic Receptor-Based Immunoassay for Influenza Vaccine Quantification
Source: PLoS One. 2013 Feb 12;8(2):e55428. doi: 10.1371/journal.pone.0055428 (PMC3570553; doi:10.1371/journal.pone.0055428)

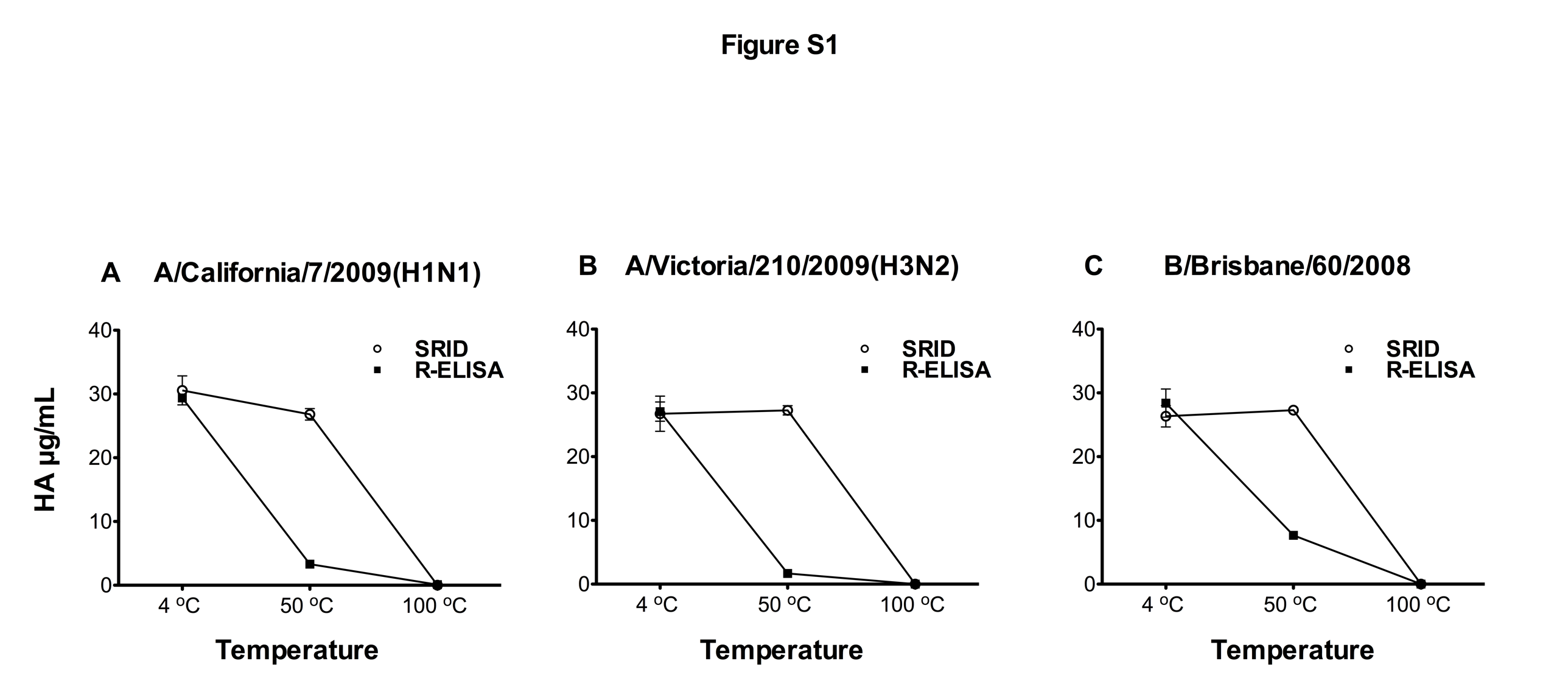

Supplement: Figure S1 — Effect of heat treatment on vaccine binding to the synthetic receptor. Influenza strains included in 2010–2011 vaccine (A) A/California/7/2009(H1N1), (B) A/Victoria/210/2009(H3N2) and (C) B/Brisbane/60/2008(Victoria-like) were incubated at 4°C, 50°C or 100°C for 1 hour then measured by both SRID and R-ELISA using the corresponding sheep strain-specific antibodies. Each treatment was tested in triplicates and experiment was repeated twice. Results are shown as mean of absorbance and error bars indicate the standard deviation. (TIFF) [file pone.0055428.s001.tiff]

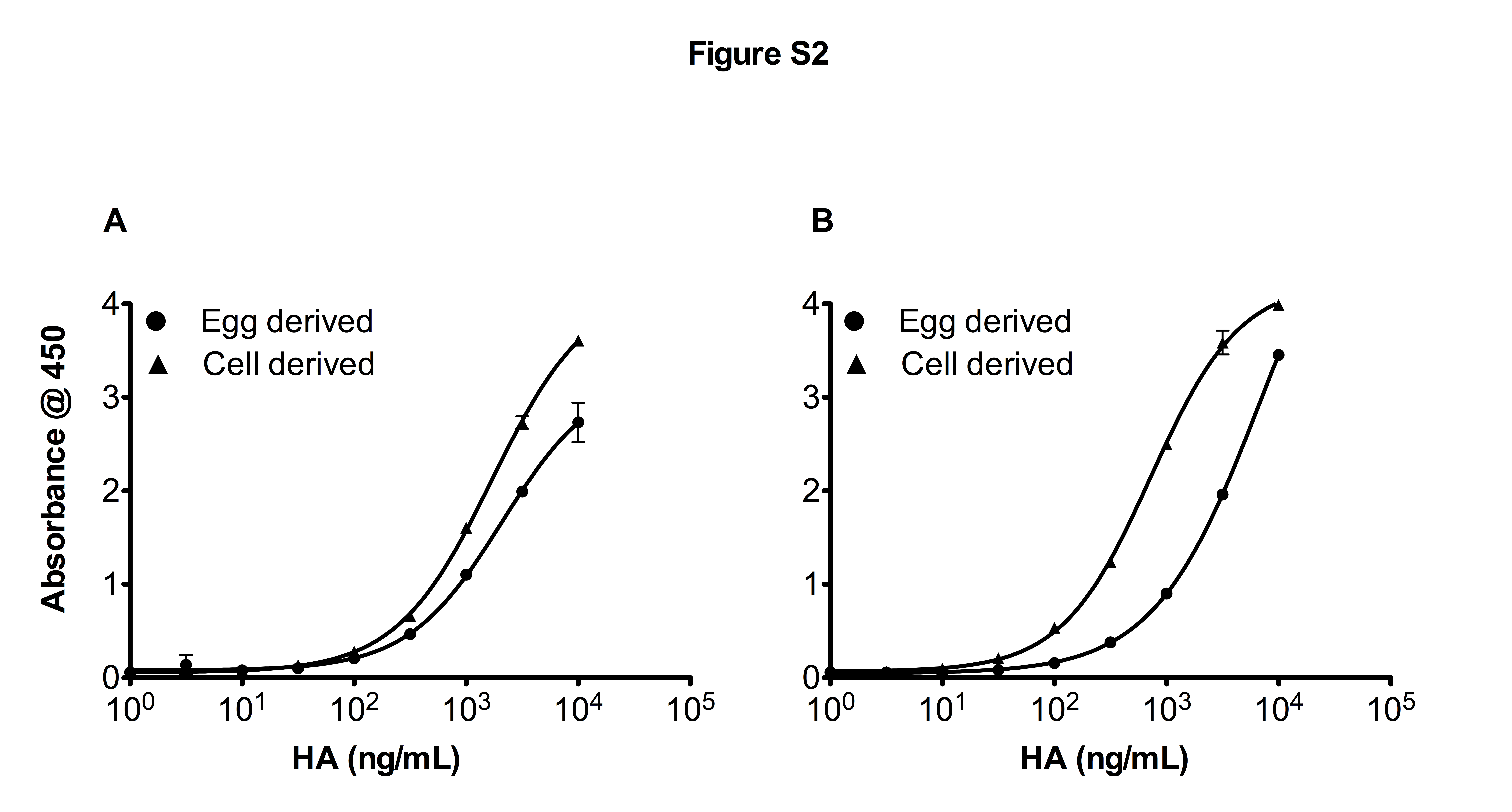

Supplement: Figure S2 — Binding of egg and cell-derived vaccines. Binding of egg or cell-derived influenza A/California/7/2009(H1N1) strain starting at 10 µg/ml was tested by the R-ELISA using the strain-specific (A) Sheep anti-HA or (B) Rabbit anti-HA Abs. Each treatment was tested in triplicates and experiment was repeated twice. Results are shown as mean of absorbance and error bars indicate the standard deviation. (TIFF) [file pone.0055428.s002.tiff]

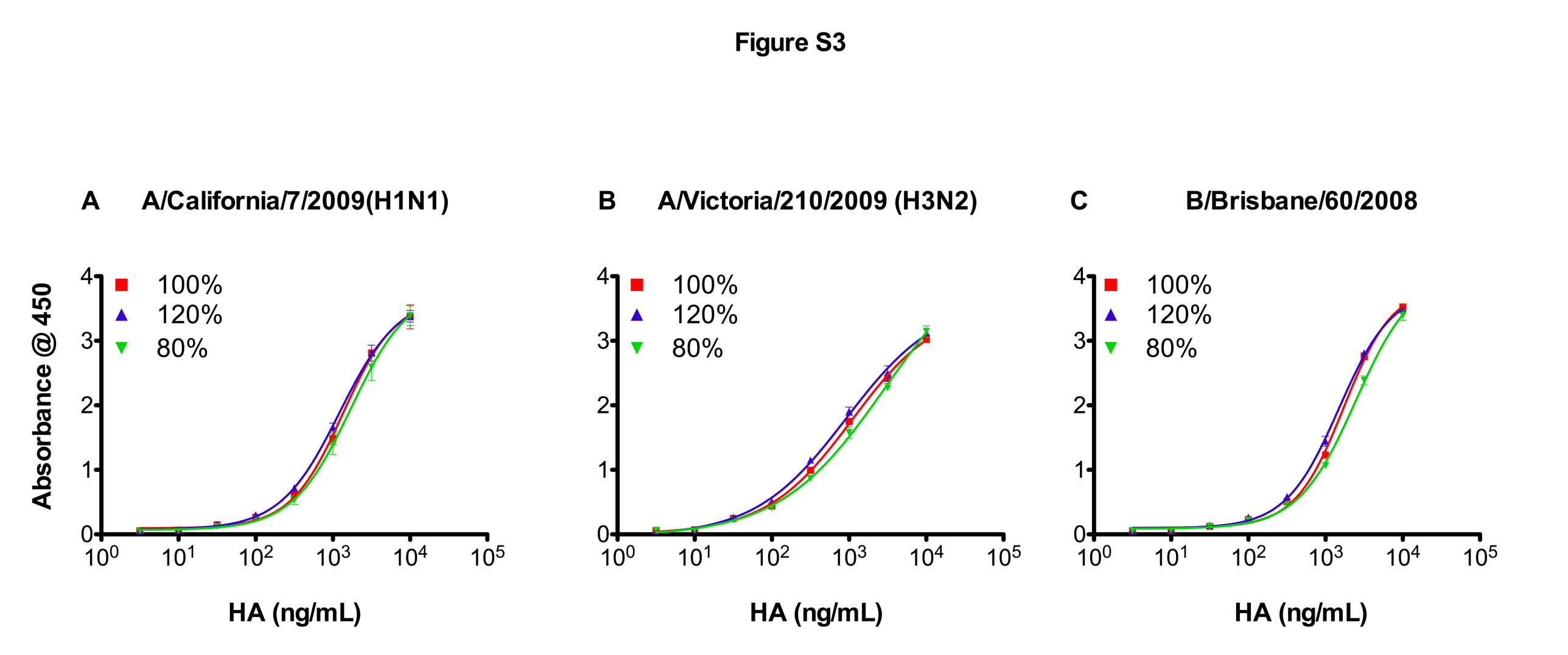

Supplement: Figure S3 — Precision of synthetic receptor based ELISA. Three expected ratios (80%, 100% and 120%) of the HA from each influenza reference strain in the 2010–2011 vaccine (A) A/California/7/2009(H1N1), (B) A/Perth/16/2009(H3N2)-like and (C) B/Brisbane/60/2008(Victoria-like) as determined by the SRID were employed in spiking and recovery assay using NIBSC sheep antisera. Each sample was tested in triplicates. Data are shown as mean +/− SD from two experiments. (TIFF) [file pone.0055428.s003.tiff]

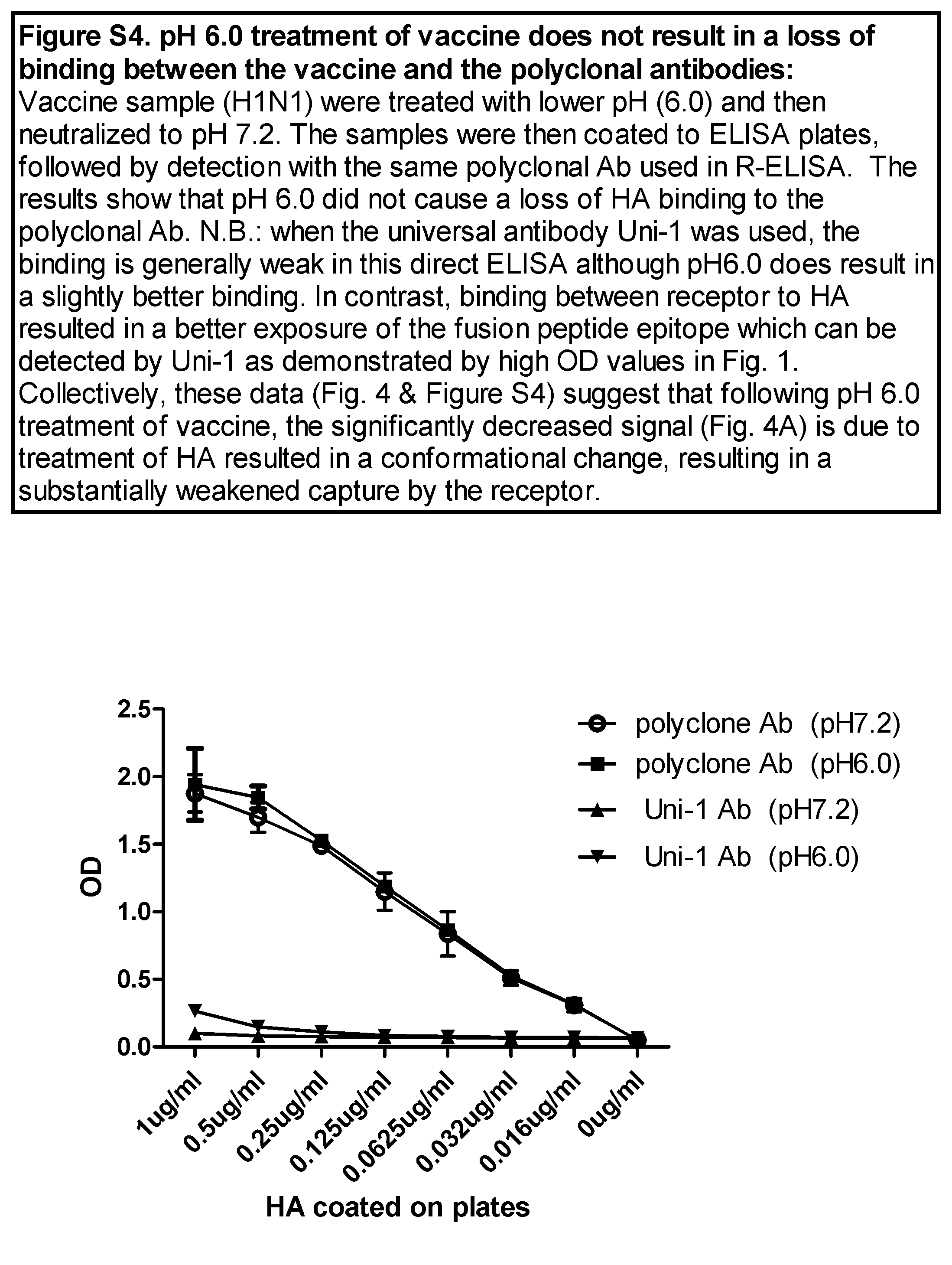

Supplement: Figure S4 — pH 6.0 treatment of vaccine does not result in a loss of binding between the vaccine and the polyclonal antibodies. Vaccine sample (H1N1) were treated with lower pH (6.0) and then neutralized to pH 7.2. The samples were then coated to ELISA plates, followed by detection with the same polyclonal Ab used in R-ELISA. The results show that pH 6.0 did not cause a loss of HA binding to the polyclonal Ab. N.B.: when the universal antibody Uni-1 was used, the binding is generally weak in this direct ELISA although pH6.0 does result in a slightly better binding. In contrast, binding between receptor to HA resulted in a better exposure of the fusion peptide epitope which can be detected by Uni-1 as demonstrated by high OD values in Fig. 1. Collectively, these data (Fig. 4 & Figure S4) suggest that following pH 6.0 treatment of vaccine, the significantly decreased signal (Fig. 4A) is due to treatment of HA resulted in a conformational change, causing a substantially weakened capture by the receptor. (TIF) [file pone.0055428.s004.tif]
